# Development and validation of a prognostic nomogram for extrahepatic bile duct adenocarcinoma

**DOI:** 10.3389/fonc.2022.950335

**Published:** 2022-11-02

**Authors:** Shiyao Zhang, You Cui, Xinyu Zhao, Qi Zhang, Chunqiang Li, Qianpeng Huang, Gang Liu

**Affiliations:** ^1^ Department of General Surgery, Tianjin Medical University General Hospital, Tianjin, China; ^2^ Tianjin General Surgery Institute, Tianjin, China

**Keywords:** nomogram, SEER, prognosis, risk factor, survival analysis

## Abstract

**Objective:**

The aim of this study is to establish a prognostic nomogram for patients with extrahepatic bile duct adenocarcinoma (EBDA).

**Methods:**

From the Surveillance, Epidemiology, and End Results database, we retrieved clinical data from 1,485 patients diagnosed with EBDA between 2004 and 2015. These patients were randomly assigned to either the training or validation group in a ratio of 2:1. Cox proportional risk regression models were used to analyze the association of each variable with overall survival (OS). Univariate and multifactorial Cox regression analyses were performed to identify prognostic factors, and prognostic nomograms were created on the basis of the results of Cox multifactorial regression analysis. Performance was assessed by calibration curves and ROC curves. Internal validation was performed using the validation cohort. The Kaplan–Meier method was used to perform log-rank constructions for different risk groups.

**Results:**

The results indicated that age, race, N and M stages of tumor–lymph node metastases based on AJCC version 6, surgery, and chemotherapy were independent prognostic factors for OS in patients with EBDA. The constructed nomograms showed decent classification in predicting both 3- and 5-year survival rates. The calibration curves also show a high degree of agreement between the predicted and actual operating systems.

**Conclusions:**

The nomogram that we constructed provides a relatively accurate and applicable prediction of survival outcome in patients with EBDA, which helps to provide reference and guidance for patient treatment.

## Introduction

Extrahepatic cholangiocarcinoma is a type of cholangiocarcinoma occurring below the hilum of the liver (1). As a relatively rare cancer, it is closely related to cholangiocarcinoma (2, 3), with poor prognosis (4) and prone to recurrence (5, 6) and metastasis (7). The incidence of extrahepatic cholangiocarcinoma shows a slight upward trend in the United States (8). Most occurrences of extrahepatic cholangiocarcinoma are adenocarcinomas (9). The assessment of cancer prognosis based on the TNM staging system is incomplete and lacks the support of demographic and clinicopathological characteristics. The prognosis of patients with extrahepatic bile duct adenocarcinoma (EBDA) is related to many clinicopathological factors, such as surgery and adjuvant chemotherapy, histological grade, and intraoperative blood transfusion (9, 10). A study has shown that pathologically poorly differentiated EBDA leads to an increased risk of lymphatic metastasis, resulting in poor prognosis of patients (6). Results of a single-center study of 83 patients with extrahepatic cholangiocarcinoma suggest that age, surgical resection, chemotherapy, and comorbidities are significant prognostic factors; postoperative adjuvant chemotherapy improves patient survival; and absence of comorbidity and presence of dysplasia are good prognostic factors (11). For patients with EBDA, there is currently a lack of effective prognostic models combining clinicopathological information to assess patients’ overall survival (OS).

Nomogram is a practical statistical tool for predicting the survival of individual patients. Studies have shown that nomogram has good accuracy in predicting the survival of tumor patients (12), and its prediction effect is better than that of a TNM staging system (13). By constructing the prognostic nomogram of patients with EBDA, the survival risk of patients can be evaluated, and the treatment plan can be adjusted for patients with high risk to enhance the treatment effect and prolong the OS of patients. In this study, we used the Surveillance, Epidemiology, and End Results (SEER) database to retrieve and collect demographic and clinicopathological characteristics, as well as treatment information, of patients with EBDA from 2004 to 2015 and constructed a nomogram to predict the prognosis of patients with EBDA, as well as conduct internal validation and clinical benefit analysis. The nomogram provides a reference for clinical diagnosis and treatment of EBDA and helps clinicians to formulate an effective treatment plan.

## Patients and methods

### Patient selection

All patient information was collected from the SEER database (https://seer.cancer.gov) and contains patient radiotherapy information [Incidence - SEER 18 Regs Custom Data (with additional treatment fields), November 2020 Sub (2000–2018)], and we downloaded the data using SEER*STAT software (release date: 8 April 2022, version 8.4.0; http://seer.cancer.gov/seerstat). Inclusion criteria were as follows: (1) primary site: extrahepatic bile duct; gallbladder cancer cases were excluded in the case-screening stage; (2) histological type: adenocarcinoma; and (3) year of diagnosis: 2004–2015, to ensure uniform TNM staging criteria. Exclusion criteria were as follows: (1) race unknown (n = 1); (2) marital status unknown (n = 56); (3) TX (n = 64); (4) NX (n = 30); (5) MX (n = 9); (6) surgery unknown (n = 2); (7) tumor size unknown (n = 871); and (8) survival time <30 or unknown (n = 251).

### Variables defined

The pre-specified variables were as follows: age, race, sex, marital status, tumor size, TNM stage based on the AJCC sixth edition, surgery, radiation, chemotherapy, vital status, and survival month.

We processed some variables in the SEER database to facilitate data analysis. Age and tumor size were transformed from continuous variables to categorical variables: age <50, 50–70, and >70; and tumor size <2, 2–5, and >5 cm. Sex was classified as men and women; race as white, black, and other races; and marital status as married and SDW (separated, divorced, widowed, or single). We collected accurate information about the TMN system based on the sixth edition of the AJCC staging.

### Statistical analysis

Our research is an EBDA prognostic model study. We included all eligible cases in the total analysis cohort and then divided them into training and validation cohorts in a 2:1 ratio, and for each categorical variable, the number and proportion of cases in each category were calculated in both data cohorts. Univariate Cox regression analysis was used to identify potential prognostic factors in the training cohort, and indicators with P-values less than 0.05 were eliminated and incorporated into the multivariate Cox proportional risk regression model. All results were represented by a hazard ratio and a 95% confidence interval. The indicators with P-values less than 0.05 were selected to construct nomograms to predict the probability of survival at 1, 3, and 5 years in the construction cohort. The area under the curve (AUC) was calculated by receiver operating characteristic (ROC) analysis for the training and validation cohorts at 1, 3, and 5 years to assess the performance of the model (AUC > 0.7 indicates satisfactory identification performance) and the agreement between the predicted and actual results of survival time. Calibration plots were drawn to evaluate the calibration ability of the nomogram. Decision curve analysis (DCA) was performed on the training and validation cohorts, demonstrating that the nomogram has a high clinical utility. We calculated the score of each patient in the model, combined with the OS time, drew the survival curve by X-tile software, and cut each risk group. Statistical analysis was performed using SPSS 22.0 software, all statistical constructions were two-sided, and P < 0.05 was considered statistically significant.

## Results

### Patient characteristics

A total of 1,485 patients with extrahepatic biliary adenocarcinoma were included in this study, of which 990 were selected in the construction cohort and 495 in the validation cohort. Patients in the training cohort were mainly aged 50–70 years (483 cases, 48.8%), and the number of patients was dominant in men (558 cases, 56.4%) and married (603 cases, 60.9%). The majority of the patients were white (792 cases, 80.0%) in terms of race, most patients (660 cases, 66.7%) had undergone surgery, fewer patients (286 cases, 28.9%) had received radiotherapy than those who had not, and fewer patients received chemotherapy (463, 46.8%) than those who did not. The baseline demographic and clinicopathological characteristics of the study population are shown in **Table 1**.

**Table 1:** Demographics and clinical characteristics of the EBDA training and validation cohorts from the SEER database.

Characteristic	All patients	Training cohort	Validation cohort	P value
	(n = 1485) No. (%)	(n = 990) No. (%)	(n = 495) No. (%)	
Age				0.5730
<50 years 50-70years >70years	82 (5.5) 718 (48.4) 685 (46.1)	58 (5.9) 483 (48.8) 449 (45.4)	24 (4.8) 235 (47.5) 236 (47.7)	
Race				0.6076
Black Others White	114 (7.7) 194 (13.1) 1177 (79.3)	73 (7.4) 125 (12.6) 792 (80.0)	41 (8.3) 69 (13.9) 385 (77.8)	
Sex				0.4147
Female Male	637 (42.9) 848 (57.1)	432 (43.6) 558 (56.4)	205 (41.4) 290 (58.6)	
Marital status				0.2732
Married SDW	919 (61.9) 566 (38.1)	603 (60.9) 387 (39.1)	316 (63.8) 179 (36.2)	
AJCC T				0.7180
T1 T2 T3 T4	330 (22.2) 277 (18.7) 582 (39.2) 296 (20.0)	218 (22.0) 191 (19.3) 390 (39.4) 191 (19.3)	112 (22.6) 86 (17.4) 192 (38.8) 105 (21.2)	
AJCC N				0.4334
N0 N1	876 (59.0) 609 (41.0)	591 (59.7) 399 (40.3)	285 (57.8) 210 (42.4)	
AJCC M				0.9540
M0 M1	1315 (88.6) 170 (11.4)	877 (88.6) 113 (11.4)	438 (88.5) 57 (11.5)	
Surgery				0.6964
No Yes	490 (33.0) 995 (67.0)	330 (33.3) 660 (66.7)	160 (32.3) 335 (67.7)	
Tumor size				0.2896
<2cm 2-5cm >5cm	542 (36.5) 837 (56.4) 106 (7.1)	375 (37.9) 545 (55.1) 70 (7.1)	167 (33.7) 292 (59) 36 (7.3)	
Radiation				0.5148
No/Unknown Yes	1064 (71.6) 421 (28.4)	704 (71.1) 286 (28.9)	360 (72.7) 135 (27.3)	
Chemotherapy				0.5315
No/Unknown Yes	799 (53.8) 686 (46.2)	527 (53.2) 463 (46.8)	273 (54.9) 223 (45.1)	

### Construction of the nomogram

**Figure 1:**
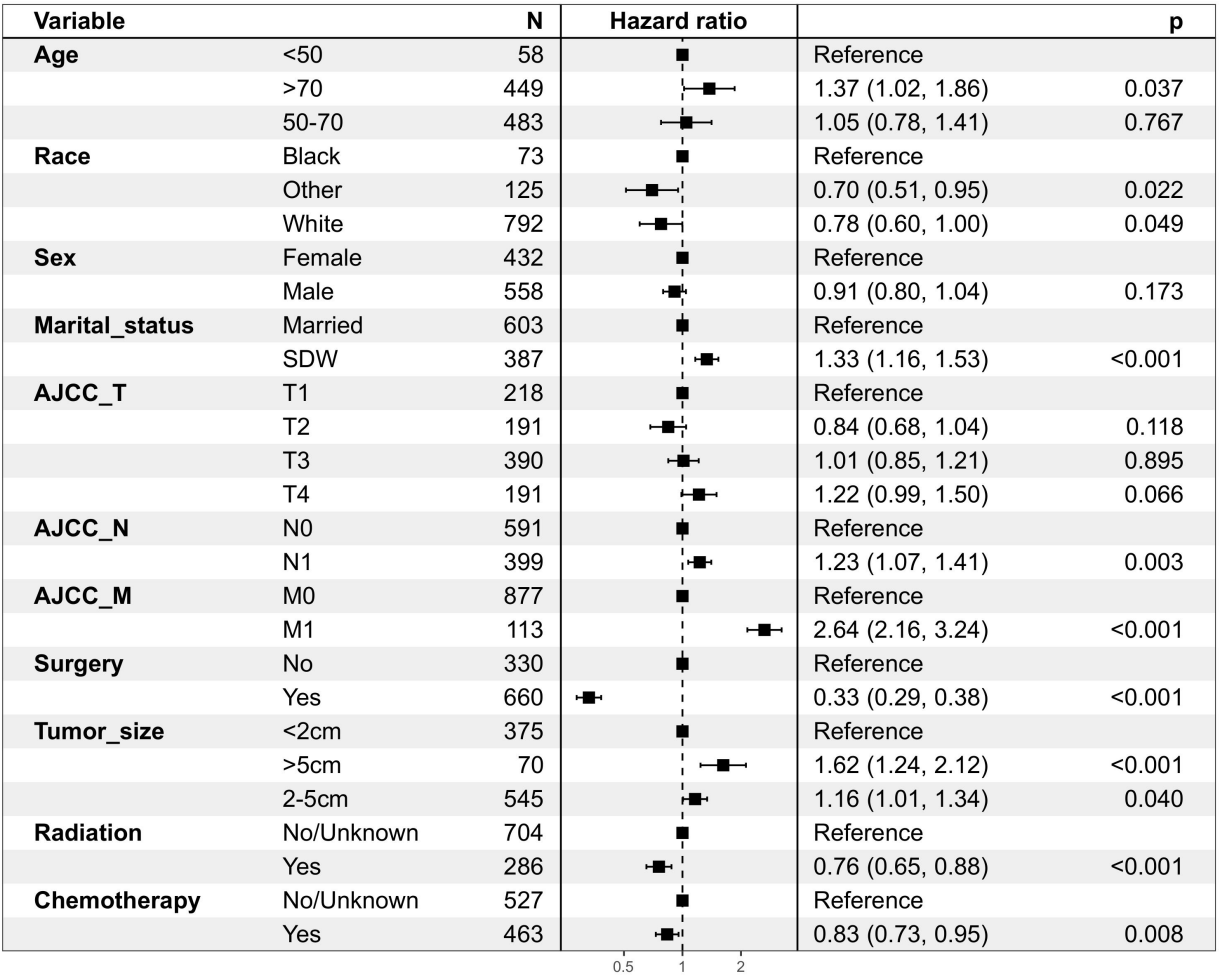
Patient and tumour characteristics and the univariate analysis of these factors on DSS (hazard ratio ± 95% confidence interval)in OS group.

**Figure 2:**
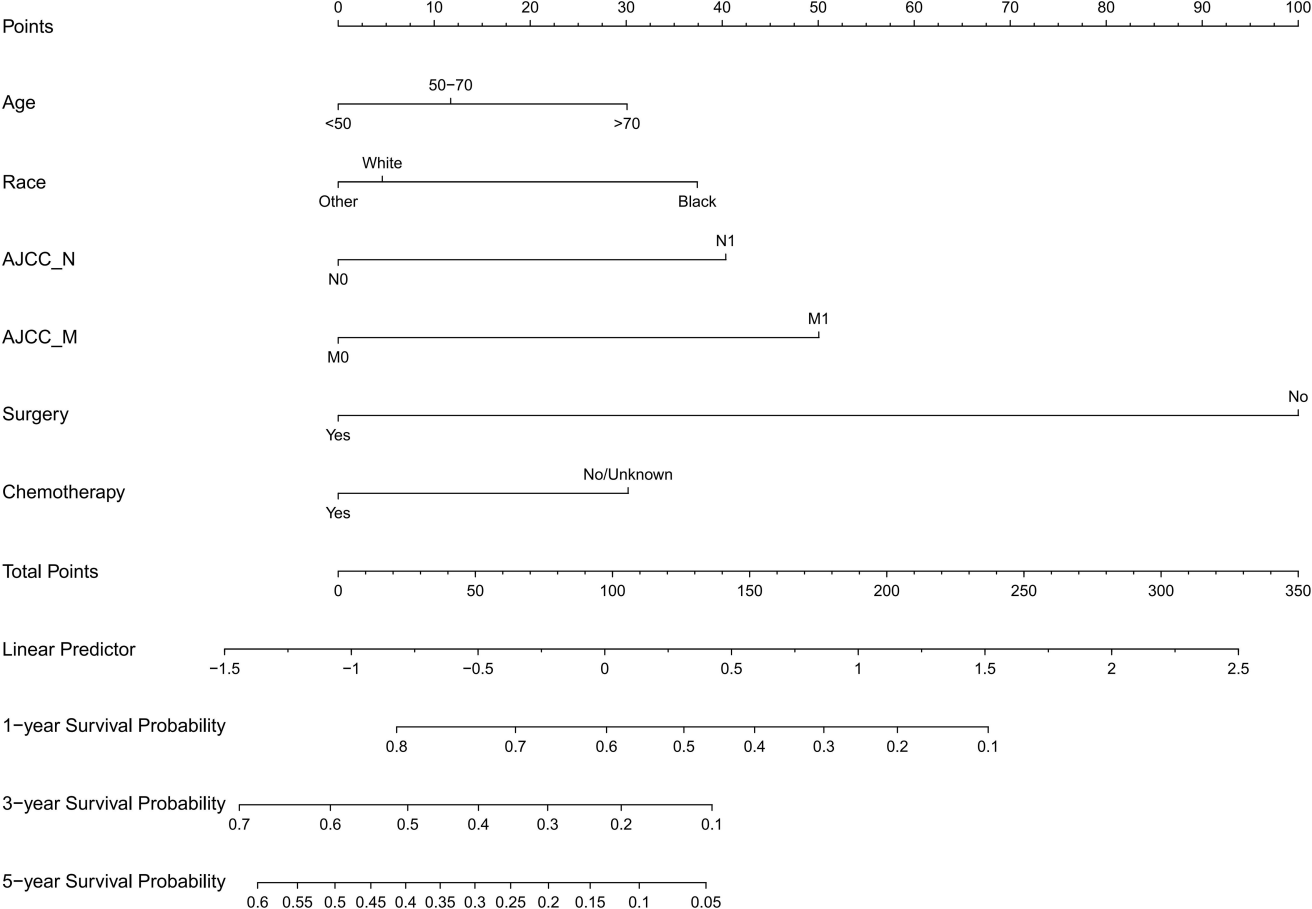
Nomogram for predicting 1-, 3-, and 5-year overall survival rates for EBDA patients.

The following potential prognostic factors were screened out by univariate Cox analysis of the training cohort data: age, race, marital status, N stage, M stage, surgery, tumor size, radiation, and chemotherapy (**Figure 1**). Six significant independent prognostic factors, namely age, race, N stage, M stage, surgery, and chemotherapy, were screened out by multivariate Cox analysis of these indicators (**Table 2**), and a prognostic nomogram (**Figure 2**) was constructed according to these factors. Nomograms predicted OS at 1, 3, and 5 years in patients with EBDA. On the basis of their contribution to the nomogram, all variables are assigned a score ranging from 0 to 100. The total score for each patient was obtained by adding the scores of each subgroup, of which the three factors with the greatest impact on prognosis were as follows (in order): no surgery, M1 stage, and N1 stage.

**Table 2:** Multivariate analyses of overall survival in the training cohort.

Variable	Multivariate analysis	
	HR (95% CI)	P value
Age		
<50 years 50-70years >70years	Reference 1.161 (0.854-1.578) 1.423 (1.038-1.950)	0.340 **0.028**
Race		
Black Others White	Reference 0.698 (0.509-0.956) 0.727 (0.562-0.942)	**0.025** **0.016**
Marital status		
Married SDW	Reference 1.138 (0.987-1.311)	0.075
AJCC N		
N0 N1	Reference 1.524 (1.314-1.767)	**<0.001**
AJCC M		
M0 M1	Reference 1.637 (1.293-2.072)	**<0.001**
Surgery		
No Yes	Reference 0.363 (0.308-0.428)	**<0.001**
Tumor size		
<5cm 5-10cm >10cm	Reference 1.051 (0.908-1.215) 1.280 (0.968-1.693)	0.505 0.083
Radiation		
No/Unknown Yes	Reference 0.909 (0.757-1.092)	0.308
Chemotherapy		
No/Unknown Yes	Reference 0.777 (0.652-0.926)	**0.005**

### Validation of the nomogram

**Figure 3:**
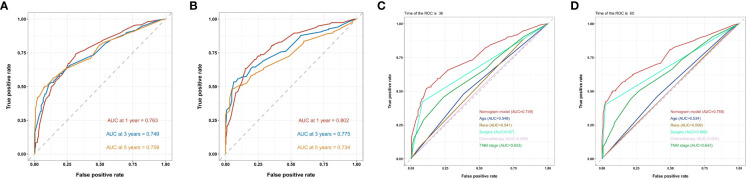
ROC curves of the nomogram predicting 1-year,3-year and 5-year OS in the **(A)** training chort of the nomogram ; **(B)** validation cohort of the nomogram ; **(C)** 3- and **(D)** 5-year ROC curve compared between the prognostic model of the training cohort and the TNM staging model and other prognostic factors in predicting OS.

**Figure 4:**
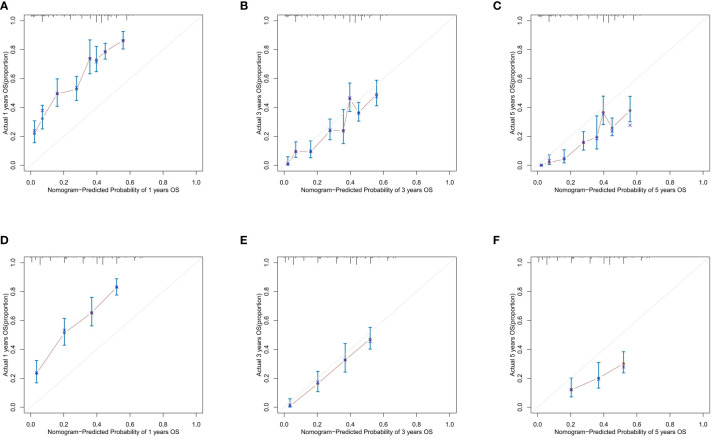
Calibration plots of the nomogram describing 1-year,3-year and 5-year OS in the training cohort **(A-C)** ; validation cohort **(D-F)**.

In the training cohort, the AUC values of 1-, 3-, and 5-year OS curves were 0.763, 0.749, and 0.759 (**Figure 3A**), respectively, and, in the verification cohort, were 0.802, 0.775, and 0.734 (**Figure 3B**), respectively. The AUC of 1-, 3-, and 5-year OS has good differentiation. We also plotted the AUC to construct the nomogram according to TNM staging; in the training cohort, the AUC values of 3- and 5-year OS curves were 0.633 (**Figure 3C**) and 0.643 (**Figure 3D**), respectively, showing that our nomogram model was superior to the model based on the TNM staging system. By drawing the calibration curves of the training cohort (**Figures 4A–C**) and the verification cohort (**Figures 4D–F**), the nomogram was verified internally. In **Figure 4**, the X-axis represented the predicted survival probability of nomogram, the Y-axis represented the actual survival probability, and the dotted line (45° diagonal) represented that the actual probability was completely consistent with the predicted probability. The results showed that the nomogram had good prediction ability. Among them, the 3-year OS prediction is the most accurate and consistent, whereas the 1-year OS prediction is slightly less accurate.

### Decision curve analysis

**Figure 5:**
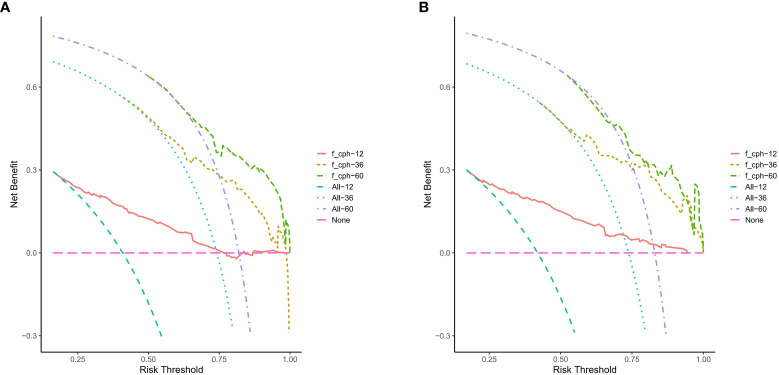
The nomogram of the Decision curve analysis in the prediction of the OS of patients at the 1-, 3- and 5-year point in the training cohort **(A)** and validation cohort **(B)**.

DCA was performed at 1, 3, and 5 years of OS in the training cohort (**Figure 5A**) and the validation cohort (**Figure 5B**). The nomogram showed a good clinical benefit in both data cohorts, with a higher clinical utility value in predicting OS at 3 and 5 years and slightly worse at 1-year OS.

### Survival curve for nomogram

**Figure 6:**
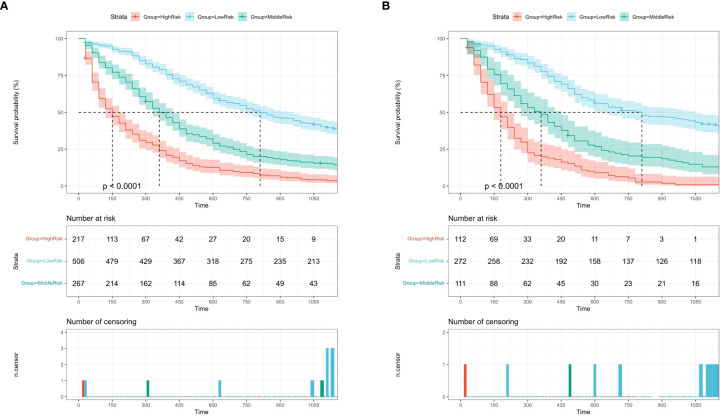
Kaplan-Meier survival curves of the training cohort **(A)** and the validation cohort **(B)** by the score calculated by the nomogram.

All variables in the nomogram are assigned points based on their contribution to the OS. Patients in the training cohort (**Figure 6A**) and the validation cohort (**Figure 6B**) were divided into three risk subgroups according to their total scores. Patients in the training cohort were divided into the following: low-risk group, <80 points; medium-risk group, 80–157 points; and high-risk group, >157 points (**Supplementary Figure S1A**). Patients in the validation cohort were divided into the following: low-risk group, <90 points; medium-risk group, 90–157; and high-risk group, >157 points (**Supplementary Figure S1B**). As shown in **Figure 6**, significant differences were observed between each of the three risk subgroups in the two cohorts (P < 0.001).

## Discussion

On the basis of the SEER database, we collected demographic and clinicopathological parameters and treatment information of 1,485 patients with EBDA. A total of 11 indexes including gender, age, race, marital status, AJCC sixth edition tumor metastasis (TNM) stage, surgery, tumor size, radiation, and chemotherapy were included. Age, race, N stage, M stage, surgery, and chemotherapy were screened as independent factors affecting the prognosis of patients with EBDA, and a nomogram (**Figure 2**) was constructed and verified. The results demonstrated that the nomogram had good accuracy and clinical benefit ability and that it could be used to guide the treatment of patients with EBDA.

According to the results of the study, age has a significant impact on the prognosis of patients with EBDA, with patients over the age of 70 having a worse prognosis. There are more male patients with EBDA than female patients, but gender difference does not constitute an independent factor of prognosis. In terms of race, there are far more white people with EBDA than other races; some studies have shown that Hispanic white people in the United States have the highest incidence of extrahepatic cholangiocarcinoma (14, 15), but according to our findings, black patients have a worse prognosis. Studies have shown that married patients with cancer tend to have a better prognosis than SDW patients (16–18); our univariate Cox analysis produced comparable results (P < 0.05), indicating that family emotional support has a positive effect on the prognosis of cancer patients. However, the results of multivariate Cox analysis showed that marital status was not an independent prognostic factor for patients with EBDA (P > 0.05); as a result, it was excluded from the nomogram model construction.

N stage and M stage were included in our nomogram construction, and T stage was excluded for lack of statistical significance by univariate Cox analysis (P > 0.05). Distant metastasis was an important factor influencing prognosis, which is consistent with another study (7). In our study, surgery proved to be an important independent factor influencing prognosis, which is in line with other studies (19, 20). Our statistical analysis shows that chemotherapy is an independent factor affecting prognosis, which is backed up by other studies (11, 21); a single-center study (11) of 83 extrahepatic cholangiocarcinoma patients reported that the median OS of all enrolled patients was 30.9 (25.1 to 36.6) months, and patients were divided into two subgroups based on surgery. Median survival was 38.5 (32.1 to 45.0) months in the surgery group and 9.9 (7.0 to 12.9) months in the non-surgery group (P < 0.05); median survival was 42.9 (34.8–51.0) months in the adjuvant chemotherapy group, 30.9 (21.8–40.1) months in the surgical chemotherapy group, 12.0 (10.3–13.8) months in the palliative chemotherapy group, and 8.9 (5.5–12.5) months in the supportive treatment group (P < 0.05), in terms of chemotherapy, indicating that patients who received chemotherapy had significantly better OS than those who did not. However, because the P-value of univariate Cox analysis was less than 0.05, radiation had no independent prognostic value. This may be due to incomplete registration of radiation information in the SEER database or selection bias due to the fact that we eliminated hundreds of cases with incomplete information. Because of the lack of detailed information about patients’ radiotherapy in the SEER database, our study is limited, and the influence of radiotherapy on EBDA needs further study. The tumor diameter showed statistical significance in univariate Cox analysis but was excluded in the construction of the nomogram because the P-value was less than 0.05 in the multivariate Cox regression model.

In recent years, through comprehensive genome sequencing of extrahepatic cholangiocarcinoma patients, including whole-genome expression, targeted DNA sequencing, and immunohistochemistry, a study (22) summarized the biological characteristics and defined the molecular typing of extrahepatic cholangiocarcinoma, providing a basis for targeted therapy. However, the relationship between molecular typing and prognosis remains to be further studied. In addition, because of the limitations of regional medical conditions and patients’ economic conditions, the nomogram that we constructed is still a simple and intuitive prognostic evaluation tool with obvious clinical benefits and has a high clinical application value.

The nomogram constructed in this study showed good differentiation (**Figure 3**), accuracy (**Figure 4**), and clinical benefit (**Figure 5**) in predicting 3- and 5-year survival. By drawing the ROC curve and calculating and comparing the AUC values, the results show that the nomogram that we constructed has better performance than the TNM staging prediction model. In addition, according to the model total scores of patients in the training cohort and the validation cohort, patients in the two cohorts were divided into high-, medium-, and low-risk groups for survival analysis (**Figure 6**). The results showed significant differences in the three groups, which also has a clinical guiding significance. The treatment plan could be adjusted according to patients’ grouping to improve the prognosis of patients. For patients with low scores, conventional treatment measures can be taken, and, for patients with high scores, intensive treatment measures are recommended.

Our study still has limitations. First, as a rare cancer, only a few cases of EBDA are included in the SEER database. Second, because of the incomplete information of some patients, we eliminated hundreds of medical records, which may lead to selection bias. Third, the SEER database index number is limited; there is a lack of some known EBDA pathological prognostic factors and testing information in EBDA genetic studies, such as the prognosis of patients with certain genetic mutations; and access to the SEER database covers a range of the US population, and differences in ethnic composition factors exist in different countries. It may be less applicable in other countries or regions. Finally, because of the rarity of EBDA and the small number of patients admitted to our hospital, external verification cannot be carried out.

## Conclusion

Our study showed that age, race, N stage, M stage, surgery, and chemotherapy were significantly correlated with the OS of patients with EBDA and were independent factors affecting prognosis. The nomogram constructed according to these factors showed good accuracy and differentiation in internal verification, which can help physicians evaluate the survival risk of patients with EBDA in clinical practice and provide a reference for treatment strategies.

## Data availability statement

Publicly available datasets were analyzed in this study. This data can be found here: SEER Database.

## Ethics statement

Ethical review and approval was not required for the study on human participants in accordance with the local legislation and institutional requirements. Written informed consent for participation was not required for this study in accordance with national legislation and institutional requirements.

## Author contributions

SZ worked on the design and conceptualization of the project and wrote the first draft. SZ and YC revised the paper. SZ and XZ conducted the statistical analysis. QZ, CL, and QH assisted in the data processing. GL, as the guarantor, proposed the concept of the project and revised the draft paper, and was responsible for the supervision. All authors contributed to the article and approved the submitted version.

## Funding

This work was funded by Tianjin Key Medical Discipline (Specialty) Construction Project.

## Acknowledgments

We are very grateful to the SEER program for approving the registration and to the SEER database.

## Conflict of interest

The authors declare that the research was conducted in the absence of any commercial or financial relationships that could be construed as a potential conflict of interest.

## Publisher’s note

All claims expressed in this article are solely those of the authors and do not necessarily represent those of their affiliated organizations, or those of the publisher, the editors and the reviewers. Any product that may be evaluated in this article, or claim that may be made by its manufacturer, is not guaranteed or endorsed by the publisher.
