# Supplementary material for: Development and validation of a prognostic nomogram for extrahepatic bile duct adenocarcinoma
Source: Front Oncol. 2022 Nov 2;12:950335. doi: 10.3389/fonc.2022.950335 (PMC9668252; doi:10.3389/fonc.2022.950335)
Supplement: Supplementary file 1 [file DataSheet_1.pdf]

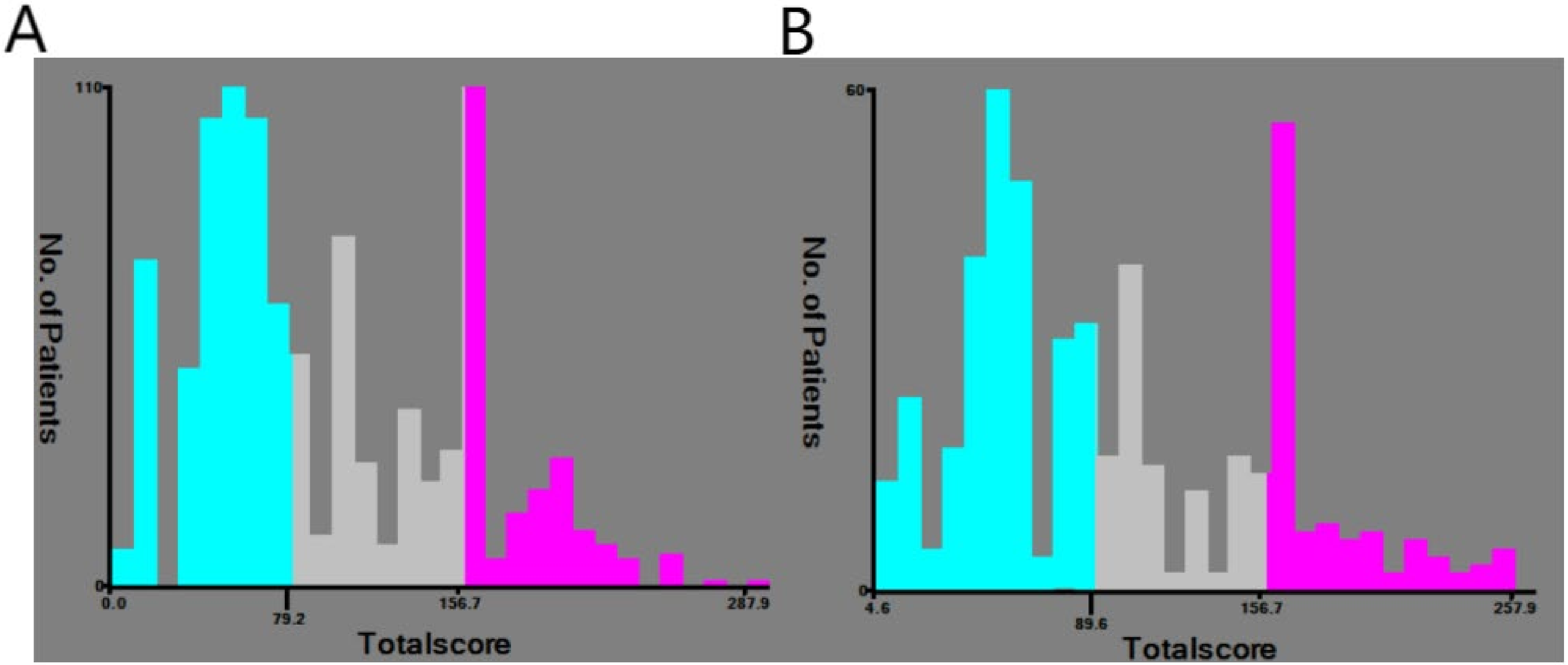

Supplementary Figure 1: X-tile divided patients from the training cohort (A) and the validation cohort (B) into risk groups of different degrees.
